# The introduction of a Parkinson’s disease email alert system to allow for early specialist team review of inpatients

**DOI:** 10.1186/s12913-019-4092-3

**Published:** 2019-04-30

**Authors:** Peter Hobson, Sally Roberts, Glesni Davies

**Affiliations:** Academic Unit, Betsi Cadwaladr University Health Board, Glan Clwyd Hospital, Rhyl, Sarn Lane, Bodelwyddan, LL18 5UJ UK

**Keywords:** Parkinson’s disease, Email alert, Hospitalisation, Length of stay, Readmission, Mortality, Economic cost

## Abstract

**Background:**

Patients with Parkinson’s disease (PWP) have complex healthcare needs, and compared to the general population, are more likely to have an unplanned emergency department (ED) attendance to hospital, along with poorer outcomes. Innovative methods of notification, when patients have an ED attendance are needed to allow for earlier specialist team interventions. This study describes the introduction of an email alert (e-alert) for a specialist Parkinson’s team. In addition, the reason for admission, specialist team interventions, length of stay, frequency of readmission, discharge destination, mortality and the bed cost per ED attendance or admission episode will be explored.

**Methods:**

The e-alert was developed in collaboration with academics, a Parkinson’s specialist team and hospital Information technology (IT) specialists, by employing existing software and IT system platforms. Patients were identified from an existing hospital patient administration and a specialist movement disorder database. Specific variables along with routine patient data were collected including demographics, clinical variables, specialist team interventions, reason for admission, length of stay, discharge destination, unscheduled readmission, mortality and bed cost per day.

**Results:**

The initial programming and setup of the e-alert was estimated to be around £3000. In its first six months, the e-alert identified 75 ED attendances, with the most common reasons being, falls and infections. The overall mean LOS was 6.8 days, with 25/75 patients being readmitted within 28 days. The most common specialist team clinical interventions were changes in medication, assessment for postural hypotension, neuropsychiatric and swallowing assessments. The majority of patients (92%) were discharged to their normal place of residence. The crude mortality rate for the cohort was approximately twice that of the hospital average. The total ED and acute bed cost was estimated to be £354,805.88, with exponential rises in healthcare costs when LOS was greater than one day.

**Conclusions:**

The Parkinson’s e-alert was found to a useful adjunct to existing hospital data systems in identifying PWP who have unplanned emergency attendances. Additionally, this system can also be employed as a service evaluation tool. However, further evaluation is needed to determine if this system can improve patient outcomes during their unplanned emergency attendance to hospital.

## Background

Urgent emergency care or unscheduled care within the National Health Service (NHS) in the United Kingdom (UK) is defined as, any unplanned contact by an individual who is seeking help, care or advice. It can consequently occur at any time and the services provided by the NHS need to be in place to meet this 24-h 365-day demand. Parkinson’s disease (PD), is strongly associated with older age, increased co-morbidly and is expected to at least double in its prevalence within the next 25 years, placing greater demands on healthcare systems [1]. Several studies have reported that patients with PD (PWP) are at a much greater risk of unplanned emergency department (ED) attendance or hospital admission compared to the general population. Furthermore, they are more likely to have longer lengths of stay, increased risk of readmission, and a higher risk of mortality [2–6]. In addition to the motor complications of PD, non-motor co-morbidities, such as, neuropsychiatric disturbance, cognitive impairment and autonomic dysfunction, are frequently reported, particularly as the disease progresses. These symptoms are often as challenging if not more so to treat, as the motor symptoms of the disease [7–9]. Earlier intervention by specialist Parkinson’s teams may have the potential to improve the outcomes for PWP who attend ED or have unplanned hospital admissions [10–12].

In view of the complex needs of PWP when they have unplanned attendances and admissions to ED or hospital, there have been calls for innovative methods of alerting specialist Parkinson’s teams when this occurs. Information technology (IT) in heath services increasingly play a vital role in the support of improving patient outcomes, and in assisting the overall management and fiscal efficiency of healthcare systems [13]. This study describes the method, introduction and economic costs of introducing an automated email alert (e-alert) system for a Parkinson’s specialist team for PWP attending ED. This study will also describe the cause for the unplanned ED attendance, the specialist team interventions, length of hospital stay (LOS), and frequency of readmission, patient discharge destination and crude mortality rate. The cost of the unscheduled ED attendance and acute hospital ward bed cost per day will also be calculated.

## Methods

The e-alert system was developed in collaboration with the academic unit, Parkinson’s team and the IT department in Betsi Cadwaladr University Health Board (BCUHB). The NHS in Wales employs a secure directory and email service, the National Active Directory Exchange system, which allows users to log onto systems regardless of where they access a computer; this can be a desktop, tablet or mobile device. This system uses existing software and IT systems and does not need any third party software to function on the existing BCUHB intranet platform. Identification of the patients for the e-alert system employs the Welsh patient administration system (WPAS) using codes G20/21 on the 10th revision of the International Statistical Classification of Diseases and Related Health Problems (ICD-10), OPCS Classification of Interventions and Procedures version 4 (OPCS-4), and a specialist movement disorder database [14, 15]. The WPAS system appends data from the specialist movement disorder database, which is a stand-alone system held upon the BCUHB web server. This database holds demographic, clinical and diagnostic information of patients with movement disorders, all of whom have been formally diagnosed by a neurologist or movement disorder specialist. In addition, all patients who attending the movement disorder clinic are routinely screen for cognitive impairment. This is based upon patient and informant review, clinical case review, neuropsychological assessment and application of DSM-5 criteria for neurocognitive disorders [16]. The patients included in this database have for example a diagnosis of probable PD based upon UKPD brain bank criteria [17]. In addition, patients with other movement disorders such as, dementia with Lewy Bodies, progressive supranuclear palsy, corticobasal degeneration, vascular parkinsonism, multiple system atrophy, etc., are also are also included in the database. The current study will only report the outcomes of the patients with a diagnosis of probable PD. The e-alert system automatically generates emails, which are distributed to the PD team (Consultant, PD nurse specialists and clinical specialist), when a patient is clerked on attendance to ED. This allows the Parkinson’s team, to view the initial ED assessment and from this, decide on the most appropriate intervention.

The development and introduction of the e-alert, along with the costs of ED attendance and acute hospital bed admission were calculated from data obtained from BCUHB finance department (May 2017). A single ED attendance cost was £191.69, and an acute ward admission (general medicine) cost was £379.52 per day. It should be noted that the acute admissions cover the daily bed cost and do not include ambulance transportation to ED, other speciality interventions, routine laboratory tests, pharmaceutical or imaging costs. Demographic details along with disease specific patient data were also recorded including, duration of PD, disease severity (modified Hoehn & Yahr staging [18]), cognitive status, time of admission, reason for admission, LOS, discharge destination, unscheduled readmission within 28 days and Parkinson’s team intervention outcomes.

### Statistics

The demographic characteristics of the patients were summarised with descriptive statistics including mean, standard deviation and median, where appropriate. In addition, the strength of linear association between variables was calculated with Spearman correlation coefficients. Shapiro-Wilk test revealed that the data was non-normal (*p < 0.001*), consequently non-parametric Mann-Whitney U analysis were used for between group comparisons. The level of significance was set at *p < 0.05*.

All analysis was performed using SPSS V22 software [19].

### Ethics

All patients gave written and informed consent to participate in this study.

The North Wales Research and ethics committee (Central) approved this study.

## Results

The initial programme writing and piloting of the e-alert was estimated to take around 50 h @ £45 per hour programming time, with an overall cost for the programming, setup and IT maintenance estimated to be less than £3000. Once established, the e-alert system requires minimal input from the IT and specialist clinical team for its maintenance. The PD team update the system electronically on a monthly basis by sending excel spreadsheets to the BCUHB informatics IT team, which include details of new cases, removal of cases due to attrition, or patients who have moved away from the BCUHB catchment area. The database costs approximately £45 per hour to update, with minimal downtime, taking on average less than three hours per month for general maintenance.

**Fig. 1:**
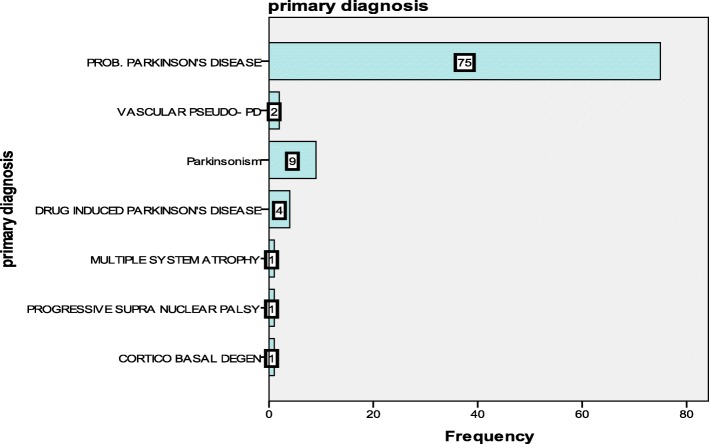
Primary diagnosis of patients (frequency) attending the emergency department (*n* = 75)

In the first six months post introduction of the e-alert, the number of patients who were captured by the system attending ED was reviewed. This revealed that 93 patients had been identified, however, upon review, 75/93 of the attendees were found to have a diagnosis of probable PD and the remaining patients had another movement disorders. The primary diagnoses of the 93 attendees to ED are given in Fig. 1. There were no differences observed in the demographics, admission or outcome data between these groups (*p* = 0.05).

The PWP attending ED consisted of 40 Male: 35 Female patients, with a mean age of 79.7 years, an age of onset of PD of 72.2 years, a median duration of PD of 6 years, and a mean modified H & Y staging of 3.6 (± 0.7). The details of which are shown in Table 1. In addition to the PD primary diagnosis, 27 (36%) had an additional diagnosis of cognitive impairment (mild cognitive impairment, 9/75 (12%): Parkinson’s disease dementia 18(24%). On admission, 13 were admitted from institutional care, 18 lived alone, with the remaining 44 patients lived with their spouse, partner or family member (Table 1). Patients who lived alone were older than those living with a partner or spouse and those living in institutional care (0.31 *p* > 0.001). The institutional care group had significantly worse PD motor (0.38, *p* < 0.001) and cognitive function compared to the other groups (0.43, *p* < 0.0001). No other differences were observed between these groups in terms of age of onset of PD, gender, LOS or mortality (*p* = 0.05). The majority of patients (69/75) were discharged to their normal place of residence. Of the remaining six patients, two entered institutional care and four (5.3%) died whilst in hospital. There were no associations found in the decedent’s age, gender, disease severity, reason for admission, or the place of residence they were admitted from (*p* = 0.05).

**Table 1:** Demographic, clinical and living status of patients (Mean ± Sd, Range, *Median*)

Age		79.7 ± 7.7	55 - 97
Gender: Male: Female			40:35
Age at diagnosis		72.2 ± 9.9	45 - 89
Duration of PD (yrs)		*6*	1 - 30
Modified H & Y		3.6 ± 0.73;	2 - 5.
Cognitive impairment	27 (36%)		
Living in institutional care	13 (17.3%)		
Living alone	18 (24%)		
Living with partner/spouse	42 (56%)		
Living with family	2 (2.7%)		

The interventions by the Parkinson’s team following an e-alert are given in Table 2. In total 69 (92%) of the patients had direct contact with a member of the specialist team. Telephone and or email advice was given to the care teams in ED in the remaining six patients who at the time were severely ill, or were at the end of life. The team reviewed each patient’s drug chart to ensure that dosage and timings were correct. Upon medication chart review, 8/41(19.5%) patients had contraindicated medications stopped or slowly titrated down to avoid withdrawal side effects. Other advice included, ensuring that the ED and acute staff were informed about the current medication and disease symptomatology of each patient. Support guidance where appropriate was given for the introduction of additional PD medications (*n* = 4), additional assessment recommendations for postural hypotension (*n* = 33), neuropsychiatric and neuropsychological assessment (*n* = 4), and safe swallowing assessment (*n* = 15). The team made interventions in the antiparkinsonian medication regime of 28(37%) patients. Nine (12%) had their medication titrated up to aid PD better symptom control. The remaining 19 patients (25%), had their PD medication reduced due to side effects including, severe dyskinesias (4/19), neuropsychiatric disturbance (3/19), confusion (9/19) and increased postural hypotension (3/19).

**Table 2:** Parkinson’s team e-alert interventions (frequencies and percentages *n* = 75)

Parkinson’s advice/review	41 (57%)
Telephone/email advice alone	6 ( 8%)
Parkinson’s medication titrated up	9 (12%)
Parkinson’s medication titrated down	19 (25%)

**Fig. 2:**
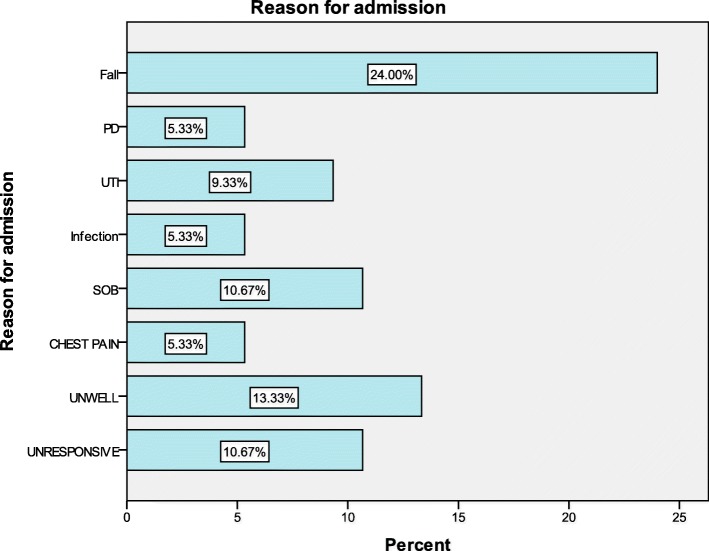
The reason for admission (%) to the emergency department

The reason for the 75 patients unscheduled attendances to ED are shown in Fig. 2. The most frequent cause was a fall (*n* = 18/75; 24%), with fractures occurring in 4/18 (22.2%).These patients were had more advanced motor (0.54, *p* < 0.0001), cognitive impairment (0.45, *p* < 0.001) and were more likely to reside in institutional care (0.41 *p* < 0.003). Around 28% (8/18) of this group, who sustained a fall, had an unscheduled readmission within 28 days of their first admission with an average additional median LOS of 13 days. Around half of the ED admissions were related to the motor and non-motor symptoms of PD, including PD medication problems, falls, neuropsychiatric problems, and urinary track and chest infections.

The mean LOS for the cohort LOS was 6.8 (± 10.8) days. However, to control for the variability in the mean LOS, the cohort was dichotomised into those who attended ED and were discharged within the same day (*n* = 23), and those who were admitted acutely to hospital from ED (*n* = 52). The first groups median LOS was 2 h.15 min (range 12 min to 24 h), with an overall bed cost of £4408.87. Patients who were admitted to an acute ward from ED, had a median LOS of 11 days (Range 2–68 days), with an overall bed cost of £354,805.88. Readmissions within 28 days of the cohort were much higher than the average of 9% in YGC (personal communication BCUHB informatics). In the current study 25/75 (33.3%) had at least one unplanned readmission and in addition to this, 28% of this group (7/25) had multiple unplanned ED readmissions, ranging from four to 12 days.

## Discussion

This study describes the introduction of an e-alert system for a specialist hospital Parkinson’s team when PWP have an unplanned attendance to ED. In addition, because this system is appended to an existing specialist database, it can be employed to evaluate the patient episodes of care for patients attending ED. Although there are initial setup costs of around £3000, when the initial programming and development stage is completed it is relatively cost neutral, because of its ease of maintenance, taking on average one to 3 h per month to update. We believe that this system can be easily employed in all hospitals with an IT infrastructure, by adapting it to suit the individual IT system platforms and clinical needs. The e-alert is flexible enough to be programmed to include alerts for elective hospital and community hospital admissions, allowing for example, specialist advice to be given to surgical or multidisciplinary teams. The limitation of this system is that many specialist teams may not have an existing database to aid identification of patients. Alternative methods of capture such as using electronic pharmacy prescribing records, movement disorder/neurology clinic lists may be necessary to develop an initial system database, although this will marginally increase the initial setup costs. This system is only useful if an assessment or intervention occurs as result of the e-alert and therefore only clinically active members of the specialist team should receive the e-alert. This avoids problems arising when too many members of a team receive emails and do not take action or responsibility for them.

How practical is this type of electronic alert system in the acute setting? Upon the introduction of the e-alert, 75 patients were identified over six months who attended ED. Within 24 h of receiving the e-alert, the Parkinson’s team were able to offer specialist advice by email, phone or direct input with the clinical teams caring for the patients. We estimate that without the e-alert, a significant number of the patients in this study would not have received this specialist team input, thus possibly delaying appropriate interventions and discharge planning advice. This is because currently patient episodes in Wales (UK) are recorded on the Patient Episode Database for Wales (PEDW) by the Welsh NHS informatics service. This is prepared by collecting patient episode data from all Welsh NHS sites monthly, which is then processed and analysed. This data for the PEDW is recorded by employing ICD10 and OPCS-4 codes and additionally includes demographic, clinical and administrative information, such as age and sex of patient and the diagnosis and operative procedures. However, the recording of ICD-10 and OPCS-4 codes only take place when a patient is discharged from a hospital. This can take from days to several months before the data is processed through the PEDW. If the PEDW had been employed alone as a method of patient capture in the current study period, we would only have been able to identify 11/75 PD cases (statistics supplied by BCUHB informatics). A partial explaination for this is that unlike other healthcare systems throughout the world, the NHS in Wales (UK) is a publicly funded healthcare system, which does not rely on for example ICD10 patient coding for reimbursement. Nevertheless, this study does highlight the weakness of this system to identify patients who may require early specialist intervention and who are potentially at a higher risk of an adverse outcome whilst attending ED in the UK.

In common with previous investigations, we found that ED attendances for PD patients were more frequently associated with falls, cognitive impairment, urinary track infections, other infections (pneumonia), and PD symptomatology [10–12]. The majority of the ED attendances in this study were directly or indirectly related to the motor and non-motor symptoms of PD. This is supported by the evidence that the cohort had advanced disease symptom severity (H & Y > 3), known autonomic dysfunction and a significant number had cognitive impairment. In view of these complex needs, early specialist opinion is essential to assist in the safe and appropriate delivery of care when patients have an unplanned ED attendance or hospital admission.

In the current study, the overall mean LOS was around seven days, which is comparable to average unplanned hospital LOS in Wales of seven days [20]. However, the crude mean values often reported most likely fail to take into account the variability and case mix in the data. Furthermore, the large standard deviations we observed in the current study would suggest that mean values reported in hospital LOS data needs to be viewed with caution. The comparison between patients attending ED and being discharged within the same day, and patients who were admitted acutely to hospital from ED in the current study, revealed that the median values are a more accurate means of describing LOS data. We also found that unscheduled readmissions within 28-days were nearly four times higher in the PD group compared to the average readmission rate in our hospital of around 9% for patients aged 55 years and over during the study period (personal communication BCUHB informatics). How typical or atypical our results for PWP are unknown, because there is a paucity of published evidence to allow for comparisons with our LOS rates. They do serve nevertheless to highlight the complex symptomatology of Parkinson’s, its treatment challenges, and the need for early specialist assessment and intervention.

Though subjective, prior to the introduction of the e-alert, the team were often called several days after admission, particularly when difficulties arose with PD symptom management, medication advice, neuropsychiatric problems or when patients were on the point of discharge. It has been estimated that the NHS in the UK spends over £200 million per year on non-elective ED admissions for PWP, with a cost per patient episode of around £3000 [12]. It is inevitable then that delays in discharge or transfer of care will substantially increase overall healthcare costs, especially as population’s age and neurological conditions such as PD become more prevalent rise. The crude bed costs presented in the current study are without doubt an underestimate of the exact costs for acute care episodes for PWP within the NHS. Despite this, the current study does provide valuable information that healthcare costs will rise exponentially if the LOS exceeds more than one day. To reduce overall healthcare expenditure, more resourceful interventions are needed, such as proactive community outreach teams or effective integration of primary and secondary care teams. Parkinson’s UK has recently proposed that improving inpatient management of PWP should be a priority within the NHS and have set out guidelines meet these challenges [21]. Whilst statements and guidelines are tentatively engaging, there is no evidence that more organised care or new service provision simply equates with reduction in healthcare costs or enhanced care. The application of more robust methodical evaluations along with sound health economic modelling is needed to determine the optimal means of introducing, building on or maintaining existing services for PD patients.

Previous studies have reported an excess mortality risk for PD patients compared to the general population when they have unplanned hospital admissions [4, 22]. The cohort in the current study had nearly a twofold increased crude mortality rate compared to the hospital (YGC) rate of around 2.6%, which is consistent with the literature [22]. Managing the complex needs of patients at the terminal stage of life is especially challenging, not only for clinical team, but also the individual, their families, friends and carers. In the UK, The National Institute for Health and Care Excellence have set out several statements and standards to address this difficult subject area as adults approach the end of their life, in the “End of Life Care, and Care of the Dying People in the Last Days of Life” documents [23]. These include, recognising when individuals are in a terminal phase of their condition, there is an open discussion with everyone involved in the patient’s life and care, planning and delivering appropriate care, recognising the physical, psychological and psychosocial needs of individuals, planning appropriate medication intervention or withdrawal. Although challenging, research is needed to determine how effective and deliverable these quality standards are, drawn from the experience of those who experience end of life care service provision.

The present and previous studies have limitations and the results should be interpreted with some degree of caution. In earlier studies, retrospective design methodology was often employed, relying upon prescribing records, hospital patient administration systems and ICD coding alone to identify patients. These methods are likely to miss a significant proportion of PD admissions and will underestimate of the true burden of this disease places upon healthcare systems [24]. The strengths of the current study are that it was able to capture the outcomes of a significant number of patients known to an existing movement disorder service. This is the first study to our knowledge that has not relied solely upon prospective or retrospective ICD-10 coding in hospital administration systems to ascertain the numbers of PWP who have unplanned attendances to ED. The e-alert is a more robust means of identifying at risk patients much earlier who have unplanned admissions to hospital. However, this type of alert will probably still not identify between 10 and 15% of admissions, because patients will not be known to the existing movement disorder service, are under other neurology services, do not have a formal diagnosis of PD or other movement disorder and come from different area health boards and/or countries.

## Conclusion

The e-alert was developed to identify and allow for earlier specialist interventions for PWP who have unplanned attendances to an ED. The system employs straightforward programming and software that is available in most IT departments within healthcare systems throughout the world. It can be introduced and maintained at modest expenditure and was found to have good user acceptance. Further evaluation is needed to ascertain if this e-alert system can assist in the delivery of timely and appropriate interventions, improve patient outcomes, reduce LOS and improve discharge planning in this complex patient group. The system described in this study was designed to be used not only as a patient alert, but also as a service evaluation tool. This is because it is linked to an existing specialist database and allows more systematic and accurate collection of data to investigate patient episodes prospectively and retrospectively than was previously possible. The results reported here support previous findings, where similar patterns of admission to hospital appear to show higher than expected mortality rates.

Further exploration with larger case controlled prospective populations are needed to fully understand why PWP have what appear to be disproportionate unplanned attendances or admission to ED or hospital, compared to the general population.

## Abbreviations

BCUHB: Betsi Cadwaladr University Health Board
e-alert: Email alert system
ED: Emergency department
ICD-10: International Statistical Classification of Diseases and Related Health Problems
IT: Information technology
LOS: Length of stay
NHS: National Health Service
PD: Parkinson’s disease
PEDW: Patient Episode Database for Wales
PWP: Patients with Parkinson’s disease
WPAS: Welsh patient administration system
